# Acquired Methemoglobinemia in an Infant: Consequence of Prolonged Application of Eutectic Mixture of Local Anesthetics (EMLA) Cream for Spontaneous Abscess Drainage

**DOI:** 10.7759/cureus.31304

**Published:** 2022-11-09

**Authors:** Abdullah Khan, Yazeed Eldos, Khalid M AlAnsari

**Affiliations:** 1 Department of Emergency Medicine, Sidra Medical and Research Center, Doha, QAT

**Keywords:** emergency, pediatric, abscess, methemoglobinemia, emla

## Abstract

Topical anesthetics are commonly used in emergency departments. One of the side effects can be methemoglobinemia if not appropriately used. We present a case of a six-week-old baby who developed methemoglobinemia with levels of 30.6% after prolonged (15 hours) application of Eutectic Mixture of Local Anesthetics (EMLA) cream. The cream was applied for spontaneous drainage of a perianal abscess. The patient received IV methylene blue with a resolution of methemoglobinemia.

## Introduction

EMLA (Eutectic Mixture of Local Anesthetics), is a water and oil-based emulsion of 2.5% prilocaine and 2.5% lidocaine [1]. The recommended dose of EMLA in children is 1 gram/10 cm^2^ of surface area applied for one hour in infants less than three months of age and for four hours in older infants and children [2]. It is commonly used in pediatric emergency departments to achieve local analgesia of intact skin for minor procedures especially intravenous cannulation, spontaneous drainage of a subcutaneous abscess, and lumbar puncture [3-5]. EMLA is a mostly safe topical anesthetic with common side effects such as mild skin reactions in the form of erythema, pallor, edema, and less common systemic side effects in the form of methemoglobinemia, and cardiovascular and central nervous system toxicity [6]. We describe one such case of methemoglobinemia secondary to prolonged application of EMLA cream in an infant. To our knowledge, previously there is no case report of acquired methemoglobinemia secondary to the application of EMLA for abscess drainage.

## Case presentation

A six-week-old male, previously healthy, was brought to the emergency department in the evening for evaluation of right-sided perianal abscess. As per history, it started as a small papule that gradually increased in size over the next five days with associated erythema and swelling but no discharge. There was no associated fever, difficulty in breathing, vomiting, or diarrhea. On examination, the patient had a 2x2 cm right-sided perianal area of erythema with a central non-draining punctum. Surgery was consulted and the plan was to apply EMLA cream for one hour with the hope of spontaneous drainage of the abscess and to return the next day morning for incision and drainage if there was no spontaneous drainage. Unfortunately, the EMLA was left on the site of the abscess overnight and the next day another dose of EMLA cream was applied. The total application time was 15 hours. The patient had no issues overnight.

The next day, on arrival at the emergency department, vitals were a temperature of 37.6 degree Celsius, respiratory rate of 42 breaths per minute, blood pressure of 86/51 millimeter of mercury, and oxygen saturation of 100% on room air. While awaiting incision and drainage the patient started to have consistent desaturations of 82% on room air with a normal waveform signal on pulse oximetry. At this point, the patient was placed on a 15 L non-rebreather face mask with minimal improvement in oxygen saturation initially. Later oxygen saturation improved to 90% on a 15 L non-rebreather face mask with the respiratory rate ranging between 27-40 breaths per minute. The EMLA cream was removed.

Examination at this point suggested normal heart sounds with no murmurs, normal femoral pulses, and bilateral normal respiratory sounds, no accessory muscle use or cyanosis. There was no family history of methemoglobinemia. Complete cell count (CBC), comprehensive metabolic panel (CMP), venous blood gas (VBG), and glucose 6 phosphate dehydrogenase (G6PD) levels were sent to the laboratory. EKG suggested normal sinus rhythm, with no hypertrophy and axis deviation. An X-ray chest suggested a normal cardiopulmonary silhouette. The VBG revealed a methemoglobin level of 30.6%, with the rest of the values unremarkable. CBC showed a hemoglobin level of 10.1 g/dl and CMP was normal. While waiting for G6PD levels, VBG was repeated in three hours which showed a methemoglobin level of 26.4%. After normal G6PD levels were reported, methylene blue was given at a dose of 1 mg/kg. The repeat VBG showed methemoglobin levels of 1.5% and the patient was successfully weaned off oxygen to room air. During this stay incision and drainage of the perianal abscess were also performed and the patient was discharged home with no complications.

## Discussion

Acquired methemoglobinemia occurs due to increased levels of oxidized hemoglobin in the blood. After exposure to oxidizing agents, the iron in hemoglobin is converted from ferrous to ferric form leading to the formation of methemoglobin which cannot bind oxygen causing hypoxemia [7]. The decreased delivery of oxygen to tissues manifests in the form of systemic toxicity such as hypoxia, central nervous system, and cardiovascular compromise [6]. The normal methemoglobin levels in the blood are less than 1.5%. The severity of symptoms depends on the level of methemoglobin in the blood with severe symptoms appearing at levels above 30% [8]. The suspicion of methemoglobinemia should be considered when hypoxia does not improve with oxygenation in a patient with no previous cardiopulmonary diseases [9]. 

Both lidocaine and prilocaine (components of EMLA) have been reported to cause methemoglobinemia but when applied on intact skin the risk of systemic absorption is low. Previously pediatric cases of methemoglobinemia with systemic toxicity have been reported after application of EMLA for circumcision, lumbar puncture, and other dermatological procedures [10]. Infants are at higher risk of developing acquired methemoglobinemia due to increased susceptibility to oxidative stress (lower NADH reductase activity) and higher levels of fetal hemoglobin (easily converted to methemoglobin) [11]. Other risk factors for systemic toxicity are related to the extended duration of application, younger age (less than three months), and inflamed skin [6]. Our patient although previously healthy had multiple risk factors such as young age, prolonged duration of application, and inflamed skin due to cellulitis and subcutaneous abscess.

Methemoglobinemia can be diagnosed by CO-oximetry (gold standard) or a combination of pulse oximetry, blood gas, and methemoglobin levels [12]. The management of acquired methemoglobinemia involves the removal of the offending agent and the administration of methylene blue (1 mg/kg) in symptomatic patients. Methylene blue can be repeated within one hour if no significant decrease in methemoglobin is noted. Methylene blue is contraindicated in patients with G6PD deficiency. In such cases, ascorbic acid can be used [13]. Our patient received one dose of methylene blue at 1 mg/kg with complete recovery and no long-term sequelae. 

## Conclusions

In summary, EMLA is a safe local anesthetic and should be used with caution in younger infants and applied with recommended doses and application time. Also, prescription of it to young infants may be avoided in order to prevent excessive use despite the medical advice. As an emergency physician, it is important to know the potential toxicities associated with topical anesthetics and the management of these toxicities.
